# Dynamics of anti-MSP3 and Pfs230 antibody responses and multiplicity of infection in asymptomatic children from southern Ghana

**DOI:** 10.1186/s13071-017-2607-5

**Published:** 2018-01-05

**Authors:** Linda E. Amoah, Festus K. Acquah, Ruth Ayanful-Torgby, Akua Oppong, Joana Abankwa, Evans K. Obboh, Susheel K. Singh, Michael Theisen

**Affiliations:** 1 Noguchi Memorial Institute for Medical Research, University of Ghana, Accra, Ghana; 2 School of Medical Sciences, University of Cape Coast, Cape Coast, Ghana; 3 Department for Congenital Disorders, Statens Serum Institut, Copenhagen, Denmark; 4 Centre for Medical Parasitology at Department of International Health, Immunology and Microbiology, University of Copenhagen, Copenhagen, Denmark

**Keywords:** Malaria, Transmission, Pfs230, MSP3, Seroprevalence, Antibodies

## Abstract

**Background:**

During a *Plasmodium* infection, exposure of human host immune cells to both the asexual and the sexual stages of the parasite elicit immune responses. These responses may be protective and prevent the development of high parasitaemia and its associated clinical symptoms, or block the transmission of malaria to an uninfected person. This study aimed at examining the dynamics of naturally acquired immune responses against the asexual and sexual forms of *Plasmodium falciparum* as well as assessing differences in the multiplicity of infection (MOI) in asymptomatic Ghanaian children living in two communities with varying malaria transmission intensities.

**Methods:**

School children aged between 6 and 12 years were recruited from Obom, a high malaria prevalence setting and Abura, a low malaria prevalence setting and enrolled in monthly multiple cross sectional surveys between February and May 2015. Filter paper blood blots (DBS) as well as thick and thin blood smears were made from finger-pricked blood at each visit. *Plasmodium falciparum* parasite prevalence was determined by microscopy and PCR. Serum eluted from the DBS were used to assess anti-Pfs230 (sexual stage) and anti-MSP3 (asexual stage) antibody levels using indirect ELISA and DNA extracted from the DBS used to assess MOI.

**Results:**

Malaria parasite point prevalence and MOI throughout the study was higher in Obom than Abura. The trend of parasite prevalence estimated by microscopy was similar to that determined by PCR in Obom but not in Abura. The trend of MSP3 antibody seroprevalence followed that of PCR-estimated parasite prevalence in Obom, while in Abura the trend of Pfs230 antibody seroprevalence followed that of PCR-estimated parasite prevalence.

**Conclusions:**

Microscopy can more accurately predict changes in parasite prevalence in high transmission settings than low transmission settings. In high transmission settings, *P. falciparum* parasite prevalence can predict antibody seroprevalence to MSP3, whilst in low transmission settings, seroprevalence against Pfs230 may be a useful predictor of parasite prevalence.

**Electronic supplementary material:**

The online version of this article (10.1186/s13071-017-2607-5) contains supplementary material, which is available to authorized users.

## Background

Asymptomatic carriage of *P. falciparum* parasites may constitute a challenge for malaria control as such individuals can serve as reservoirs for transmission [1].

Individuals living in malaria endemic regions develop partial immunity against clinical malaria after repeated exposure to *P. falciparum* [2, 3]. This immunity is at least in part mediated by IgG antibodies, as the administration of purified immunoglobulins from immune individuals to infected individuals results in reduction of parasitaemia and clinical symptoms [4]. Although this immunity effectively reduces the development of disease, it does not provide sterile protection but allows semi-immune individuals to carry parasites in the absence of clinical disease symptoms [5]. However, asymptomatic parasitaemia has been seen more in individuals in high transmission areas [6] and in those harboring multiple parasite clones [7]. Whereas anti-malarial immunity is well described, the mechanisms underlying naturally acquired immunity are not fully understood.

Some studies have shown that individuals living in areas of seasonal malaria transmission naturally acquire antibodies against multiple stages of the parasite [8, 9]. Antibodies against some asexual blood stage antigens such as the merozoite surface protein 2 (MSP2) may be highly prevalent irrespective of transmission season [10, 11], probably as a result of the persistent presence of asymptomatic parasites [12] or long-lived antigen-specific antibody responses [10]. Antibodies against others such as the sporozoite stage antigen circumsporozoite protein (CSP) have been found to be relatively short-lived and change significantly with transmission intensity [13, 14]. Antibody responses against several asexual stage antigens including the merozoite surface antigens 1 (MSP1) and 3 (MSP3), microneme proteins erythrocyte binding antigen 140 (EBA140) and 175 (EBA175) and rhoptry proteins 2 (Rh2) and 5 (Rh5) have been associated with protection from clinical malaria in children aged 5–14 years [15]. There is, however, evidence that anti-merozoite antibody titres may also be markers for increased risk to malaria [16].

The sexual stages of *P. falciaprum*, known as gametocytes, are the transmissible forms of the parasite. It is currently known that the sexual stage parasites are formed during every asexual blood stage cycle [17] and present at a fixed ratio to the asexual parasites in asymptomatic infections [18–21]. Although immune responses to antigens of this stage have been described in malaria endemic populations [22–24], the dynamics of these responses over time are not as well described as those against a number of asexual stage antigens. Immunity that develops against sexual forms of the parasite does not confer protection at the individual level but may prevent the spread of malaria in the population through blockade of parasite development in the infected mosquito [25, 26].

The dynamics of the naturally acquired antibodies against a merozoite surface antigen (MSP3) and the sexual stage antigen (Pfs230), has not been well studied in asymptomatic individuals living in different transmission settings in Ghana. This study aimed to assess the acquisition of antibodies against MSP3 and Pfs230 in two asymptomatic populations living in the southern zone of Ghana in the transition period from the dry to the major rainy season.

## Methods

### Study site and population

**Fig. 1:**
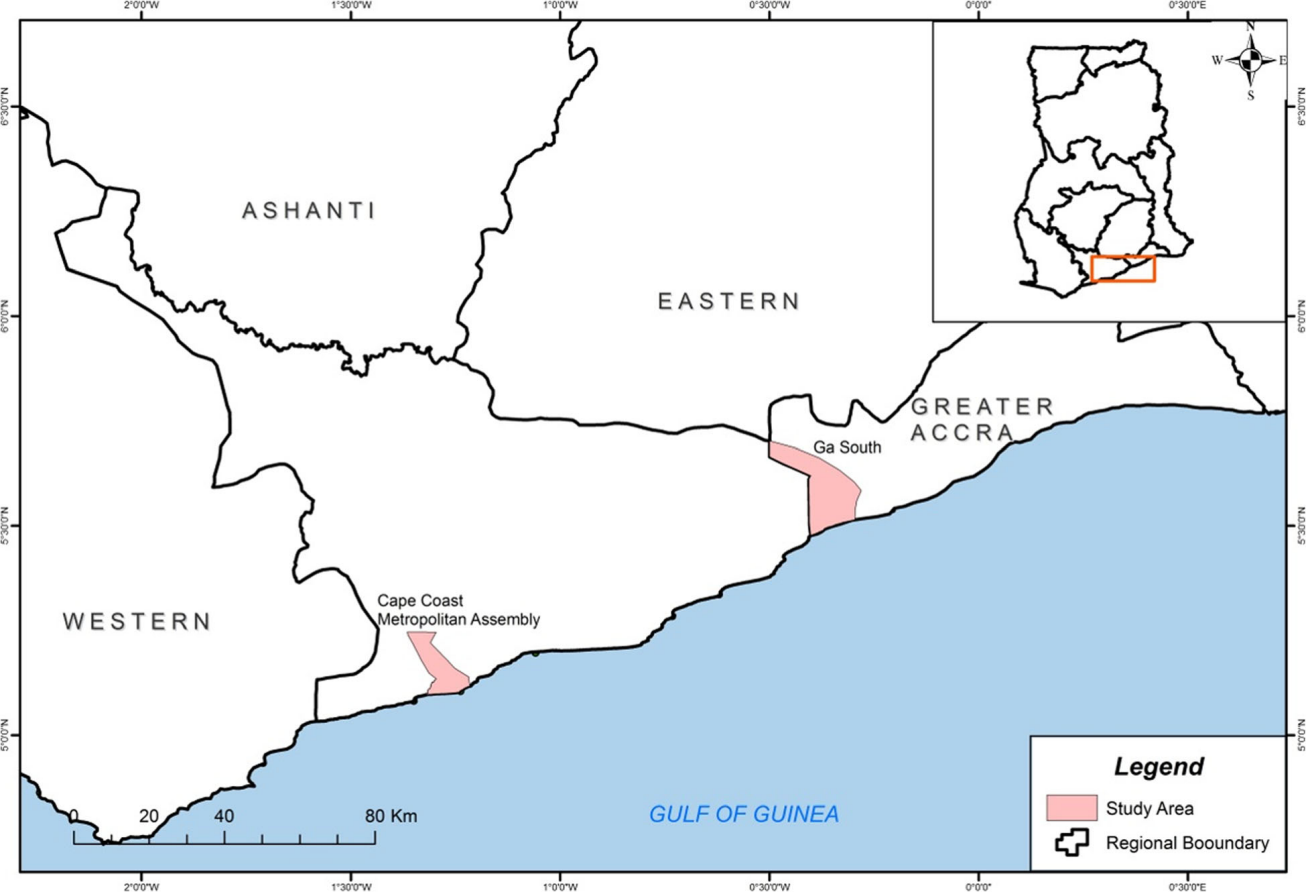
Map of Ghana highlighting the study sites. Children from a basic school in Obom in the Ga South Municipality (highlighted) and Abura in the Cape Coast Metropolitan Assembly (highlighted) of Ghana were recruited in this study

The study was conducted in two communities within Southern Ghana where the main cause of malaria is *P. falciparum*. Obom is within the Ga South Municipality of the Greater Accra Region of Ghana and Abura is in the Abura municipality of the Central Region of Ghana (Fig. 1). Malaria transmission in Abura is seasonal but perennial in Obom [27] although both sites have the peak malaria season coinciding with the major rainy season between June and August. *Plasmodium. falciparum* prevalence is, however, higher in Obom than in Abura. A total of 75 primary school children aged between 6 and 12 years without the symptoms of clinical malaria from Obom and 65 from Abura were recruited into the multiple cross sectional study.

### Sample collection and processing

Samples were collected monthly for a period of four months (February to May, 2015). During each visit, 100 μl of finger-pricked blood was collected and used to prepare thick and thin blood smears on microscope slides as well as spot on Whatman filter paper (GE Healthcare, WI, USA). The filter paper blood blot (DBS) was air-dried and stored desiccated at room temperature for less than a month.

### Estimation of parasite prevalence and density by microscopy

Each thick blood smear was air-dried and stained in 10% Giemsa for 15 min and then air-dried again [28]. The smears were then observed under ×100 oil immersion using a compound microscope and the number of *P. falciparum* infected red blood cells (iRBCs) observed after counting 200 white blood cells (WBCs) was recorded. The number of iRBCs was converted into parasite density/μl blood by multiplying the value by 40 (based on the assumption that there are 8000 WBCs/μl of blood) [29]. Two independent microscopists read each blood smear.

### Parasite genotyping

#### Extraction of parasite DNA and antibody elution from filter paper

DNA extraction was carried out by the Saponin-Chelex extraction method as previously described [30]. Briefly, two 3 mm discs were punched out of the dried filter paper blood-blots (DBS) and incubated in 1120 μl of phosphate buffered saline (PBS) containing 0.5% saponin (PBSS) overnight at room temperature on a shaking incubator. The next day, the PBSS containing the eluted antibodies was decanted into a microcentrifuge tube and used immediately for ELISA. The discs were subsequently washed twice with 1 ml PBS and then incubated in 150 μl of 6% Chelex-100 (Sigma-Aldrich, MD, USA) in DNase/RNase free water for 5 min at 95 °C. After a final high speed centrifugation step, the DNase/RNase free water containing the extracted genomic DNA was collected into a new microcentrifuge tube and stored at -20 °C until further use.

#### PCR genotyping and determination of multiplicity of infection

The *msp*2 and *glurp* genotyping reactions were performed using a protocol adapted from [31, 32] to determine the number of different parasite clones in each asymptomatic infection. Family-specific primers were used to amplify the *msp*1 and 2 genes using a nested PCR as previously described [33]. Briefly, the primary PCR reaction consisted of 200 nM dNTPs, 2 mM MgCl_2_, 133 nM of each forward and reverse primer, and 0.5 units of One Taq DNA polymerase (New England Biolabs, Hitchin, UK) in addition to 4 μl of genomic DNA (gDNA) template. The secondary reaction mixture contained 200 nM dNTP, 1.8 mM MgCl_2_, 200 nM of each forward (S1fw) and either of the allele specific reverse primers (M5rev and N5rev) and 0.5 unit of One Taq DNA polymerase supplemented with 0.5 μl of the primary PCR product. A semi-nested PCR reaction was used to amplify the *glurp* gene using a reaction mixture containing 200 nM dNTP, 2 mM MgCl_2_, 200 nM of each forward and reverse primer and 1 unit of One Taq DNA polymerase. The primary and semi-nested reaction mixtures for *glurp* differed only in the amount of template used: 4 μl of gDNA for the primary and 1 μl of primary PCR product for the semi nested reaction. Each PCR reaction contained positive controls: MRA-102G (3D7) for *msp*2 3D7 alleles, MRA-159G (KI) for *msp*1 KI and *msp*2 FC27 alleles, MRA-155G (HB3) for *msp*1 MAD20/*msp*2 FC27 alleles and MRA-200G (R033) for *msp*1 RO33/*msp*2 3D7 alleles and a no template negative control. All primers used and their annealing temperatures are listed in Additional file 1: Table S1. PCR products were separated on 2% ethidium bromide-stained agarose gels and visualized under UV illumination. Additional file 2: Figure S1 contains representative agarose gel images of 3D7 and FC27 *msp*2 allele specific PCR products from some samples collected from Obom.

#### Quantification and seroprevalence of IgG against Pfs230 and MSP3

IgG antibodies against the antigens were quantified by indirect ELISA as previously described [34] with slight modifications. Briefly, the recombinant antigens Pf230_C0Ll_ in carbonate buffer, pH 9.0 [34] and MSP3 in phosphate buffered saline (PBS, pH 7.4) [35] were used to coat NUNC Maxisorp ELISA plates at 1 μg/well and stored at 4 °C overnight. The plates were then washed four times with 250 μl/well of wash buffer (PBS containing 0.05% Tween 20 (PBS-T)). Unbound regions in the wells were then blocked with 150 μl of blocking buffer (3% skimmed milk in wash buffer) for 1 h. The plates were washed twice and incubated for 1 h with 100 μl/well eluted antibodies in duplicate. Each plate had a negative and positive control sample, obtained from a pool of antibodies eluted from individuals respectively confirmed as seronegative and seropositive to the same antigens in a previous study [34]. A calibration curve produced by serially diluting 1 mg/ml of polyclonal reference IgG three-fold was also included [35]. Plates were washed four times and incubated with 50 μl of 1:3000 dilution of rabbit antihuman IgG-HRP (Dako, DK) for 1 h. The plates were further washed and incubated with peroxidase substrate TMB (3,3′,5,5′-tetramethylbenzidine) for 30 min and the enzyme reactions stopped by addition of 50 μl of 0.2 mM sulfuric acid. The optical densities (OD) of the contents of the wells were then read using a Biotek ELISA plate reader at 450 nm.

### Data analysis

Data were entered into Excel and analysed using GraphPad Prism v.5. Data were tested for normality with the Shapiro-Wilk normality test. Mann-Whitney U-test and Kruskal-Wallis test were used to compare the differences between groups. Statistical significance was set at 0.05 unless otherwise stated. Samples that produced more than a single product after the nested or semi-nested PCR reaction were considered to harbor multiclonal infections. When no PCR product was obtained after a repeat gDNA extraction and subsequent PCR amplification, the sample was considered negative for *P. falciparum* (uninfected). ADAMSEL (Ed Remarque, BPRC) was used to convert OD values into concentration. Individuals with IgG concentrations higher than mean value for the negative (malaria naïve) control plus two standard deviations were rated seropositive. A smear was considered positive when at least one *P. falciparum* parasite was observed after counting at least 200 white blood cells.

## Results

The proportion of males to females during the entire sampling period was essentially the same for the two study sites ranging between 41.7–46.7% in Obom and between 42.4–46.3% in Abura (Table 1). The median age of participants was similar at both sites (Table 1). None of the children presented signs of clinical malaria during any of the three follow-up visits (Table 1).

**Table 1:** Demographic characteristics of participants

	February		March		April		May	
	Obom	Abura	Obom	Abura	Obom	Abura	Obom	Abura
Children, *n*	75	65	62	59	60	54	62	55
Male, *n* (%)	35 (46.7)	29 (44.6)	28 (45.2)	25 (42.4)	25 (41.7)	25 (46.3)	28 (45.2)	25 (45.5)
Median age (IQR)	9 (8–10)	9.5 (9–11)	9 (8–10)	9 (8.5–10)	9 (8–10)	10 (9–11)	9 (8–10)	9 (8.5–10)
Micro. positive	34	7	27	0	33	2	8	0
PD GM (95% CI)	441.8 (295.3–660)	321.7 (95.91–1079)	693.6 (420.2–1145)	0	712.6 (474.3–1071)	nd	155.4 (101.5–237.9)	0

*Abbreviations*: *n* number, *IQR* interquartile range of age in years, *Micro. positive* number of children positive for *P. falciparum* by microscopy, *PD* parasite density/μl; *GM (95% CI)*, geometric mean with 95% confidence interval in parentheses; *nd* only two samples were positive

**Fig. 2:**
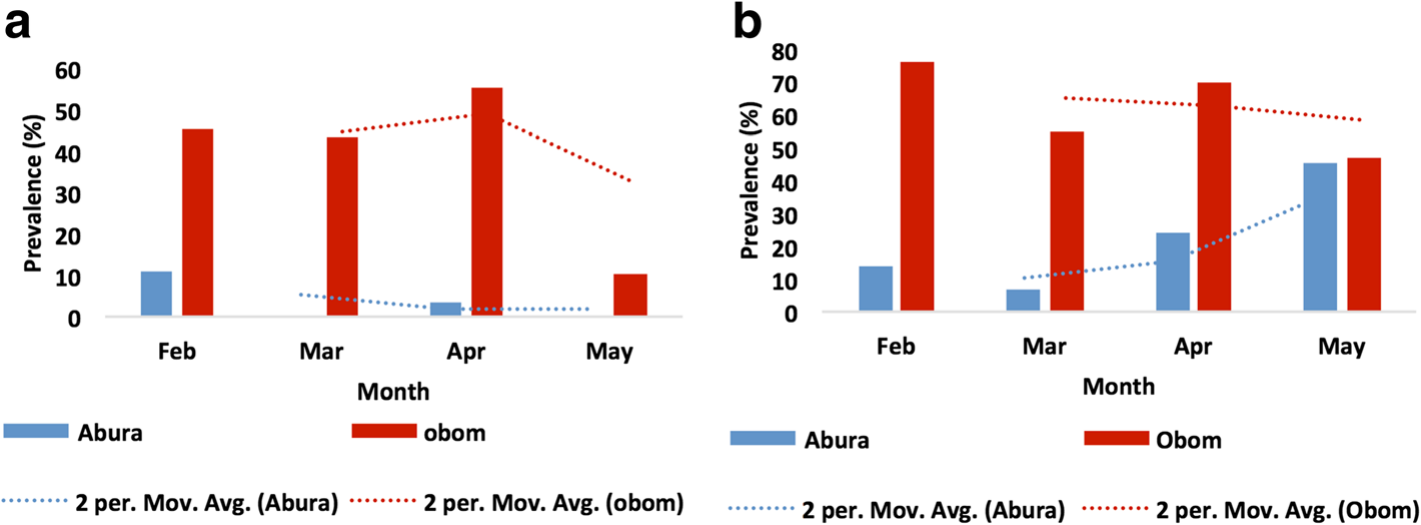
*Plasmodium falciparum* prevalence. Parasites were detected by the microscopic evaluation of Giemsa stained thick smears (**a**). PCR based MSP3/GLURP genotyping (**b**) was performed on gDNA extracted from the DBS obtained from the children living in Obom and Abura and over the four month study period

Parasite prevalence by PCR was estimated based on samples that tested positive for *P. falciparum*, by producing a PCR product after the *msp/glurp* genotyping reactions. *Plasmodium falciparum* parasites were detected by microscopy as well as by PCR and found to be prevalent throughout the study period. PCR detected higher asymptomatic parasite prevalence than microscopy in both Obom and Abura (Fig. 2a, b). Regardless of the detection method used, the prevalence of asymptomatic children was always higher in Obom than Abura during the entire study period. Asymptomatic parasite prevalence detected by microscopy in both sites appeared bimodal with the highest records of 45 and 55% for Obom and 10.8 and 3.7% for Abura occurring in February and April, respectively. PCR analysis, however, indicated that the number of asymptomatic children in Obom decreased from the initial visit through to the end of the study, whilst an opposite trend was observed in Abura (Fig. 2b). Parasite densities (PD) in both sites were generally low with geometric mean densities below 1000 parasites per microliter (Table 1). In Obom, the PD did not vary significantly from February to April (Kruskal-Wallis test, *χ*^2^ = 3.14, *df* = 2, *P* = 0.20) but decreased significantly in May (Mann-Whitney U-test, *U*_(34,8)_ = 34.5, *P* = 0.001). Statistical comparisons of PD in Abura could not be done due to the low prevalence of microscopic parasites (Table 1).

**Fig. 3:**
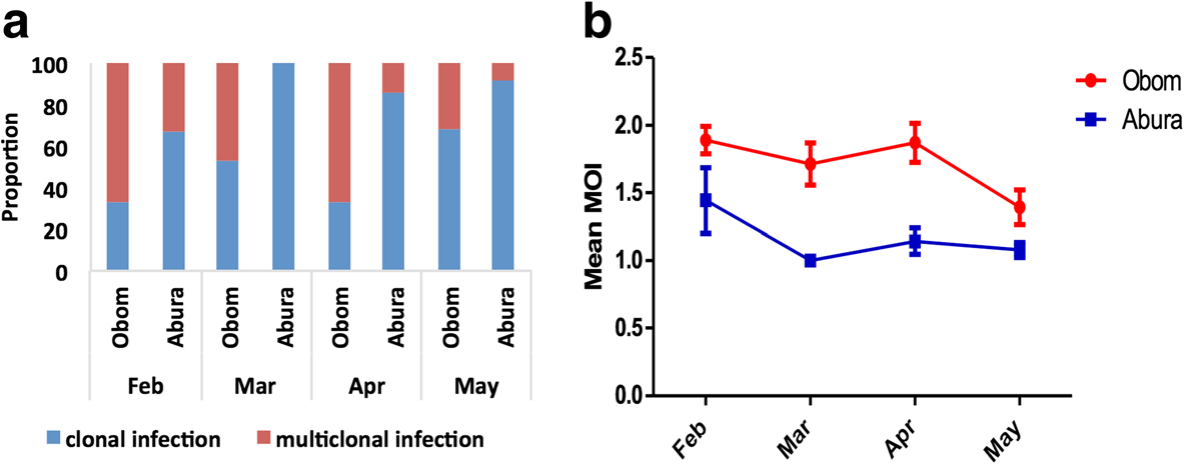
Characterization of the clonality of an infection. The variations in the proportion of single or multiple clone infections (**a**) and the geometric mean MOI in Abura and Obom over the study period (**b**). After PCR amplification of either *msp*2 or *glurp* from gDNA extracted from dried blood spots of the children, the children were grouped into categories depending on the total number of infecting parasite clones that were contained in each asymptomatic infection. The presence of either a single *msp*2 or *glurp* allele (clonal), or multiple alleles (**a**) was documented. The geometric mean MOI for all the asymptomatic children from Obom and Abura was determined for each month (**b**). The 95% confidence intervals of the GM are represented as the error bars in (**b**)

The proportion of children carrying more than one parasite clone was higher in Obom, the high transmission setting, than in Abura, where the parasite transmission is low (Fig. 3a, Table 2).

**Table 2:** Number of participants with clonal or multiple infections

	February		March		April		May	
No. of clones	Obom	Abura	Obom	Abura	Obom	Abura	Obom	Abura
0^a^	20	56	27	55	29	40	34	30
1	18	6	19	4	11	12	20	23
2	26	2	9	0	14	2	5	2
3	10	1	5	0	5	0	3	0
4	1	0	2	0	1	0	0	0
Total	75	65	62	59	60	54	62	55

^a^No PCR product was obtained after a repeat genotyping PCR. Children from whom no PCR product was obtained represent healthy children as opposed to asymptomatic children whose samples yielded PCR products

**Fig. 4:**
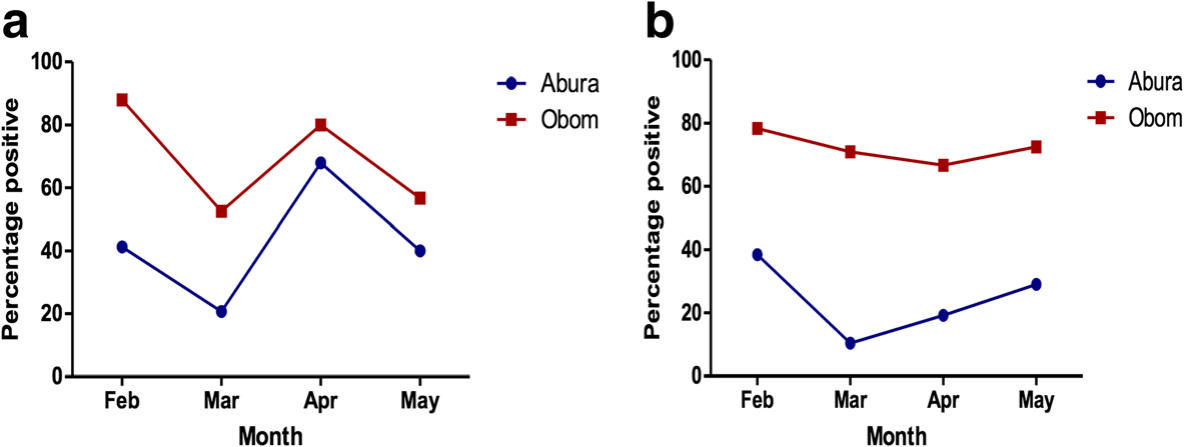
The proportion of children who were seropositive for MSP3 and *Pfs*230. Recombinant MSP3 (**a**) and Pfs230-C0_Ll_ (**b**) antigens were used to measure naturally acquired IgG responses in plasma from the children over the four-month study period (February to May 2015) using indirect ELISA. The responses were categorized as positive or negative based on antibody concentrations higher than the negative control (malaria naïve plasma) cutoff of for MSP3 and *Pfs*230-C0_LI_, respectively

Consequently, the mean MOI observed throughout the period were always higher in Obom than in Abura (Fig. 3b), although the differences observed were not significant for all months except April (Mann-Whitney U-test, *U*_(14,31)_ = 102.0, *P* = 0.0019). Seroprevalence of antibodies to MSP3 (Fig. 4a) and Pfs230 (Fig. 4b) was generally higher in Obom than in Abura during the four-month study period. Seroprevalence of antibodies against MSP3 showed marked seasonal variations over the four months of the study in both sites. However, seroprevalence of antibodies against Pfs230 showed very little variation in the high transmission setting.

**Fig. 5:**
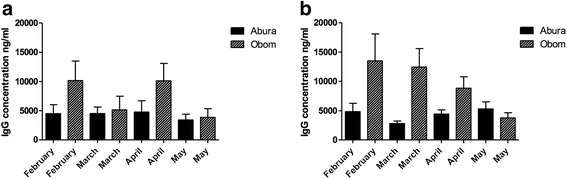
IgG antibody titres against MSP3 (**a**) and Pfs230 (**b**) over the study period. An indirect ELISA was used to test the recombinant antigens for reactivity to antibodies eluted from the dry blood blots prepared from blood collected from the children from February to May 2015. The graph represents the geometric mean of the antibody concentrations of all the children from Obom and Abura and the error bars represent the 95% confidence intervals

The geometric mean concentrations of IgG antibodies against MSP3 in children from Obom in February and April were significantly higher (Mann-Whitney U-test, *U*_(61,74)_ = 1399, *P* = 0.0002 and *U*_(51,60)_ = 830.0, *P* < 0.0001 respectively) than those in children from Abura during the same months (Fig. 5a). The geometric mean IgG levels to MSP3 in the Abura over the study period did not vary significantly (Kruskal-Wallis test, *χ*^2^ = 5.60, *P* = 0.133). In Obom, the geometric mean of IgG concentrations showed significant (Kruskal-Wallis test, *χ*^2^ = 29.14, *P* < 0.0001) sequential monthly variations with IgG concentrations in February and April being high whereas those of March and May were low. IgG concentration of antibodies against Pfs230 in children from Obom were significantly higher than those in children from Abura during the first three months, February through April (Mann-Whitney U-test, *U*_(65,74)_ = 1291, *P* < 0.0001; *U*_(58,62)_ = 232.0, *P* < 0.0001; *U*_(52,60)_ = 625.0, *P* < 0.0001, respectively), however during the last month (May) antibody concentrations were significantly higher in Abura than Obom (Mann-Whitney U-test, *U*_(55,62)_ = 1309, *P* = 0.031) (Fig. 4b). Antibody concentrations of anti-Pfs230 antibodies in children from Obom were decreased gradually over the course of the study, however it was not until the fourth month that the decrease was significant (Mann-Whitney U-test, *U*_(60,62)_ = 783, *P* < 0.0001) (Fig. 4b). The concentrations of antibodies against Pfs230 were relatively stable in Abura. There were high degrees of correlation between antibody responses to MSP3 and Pfs230 over the study period for Obom and Abura, respectively (Table 3).

**Table 3:** Spearman's rank correlation coefficients of MSP3 and Pfs230 IgG concentration in study sites over the study period

	Abura	Obom
February	0.847 (*P* < 0.0001)	0.727 (*P* < 0.0001)
March	0.770 (*P* < 0.0001)	0.522 (*P* < 0.0001)
April	0.436 (*P* = 0.0014)	0.752 (*P* < 0.0001)
May	0.630 (*P* < 0.0001)	0.741 (*P* < 0.0001)

## Discussion

In areas of seasonal malaria transmission, the highest prevalence of malaria parasites occurs at the peak of the season which follows soon after the peak of the rainy season [36, 37]. This has been attributed to an increase in vector breeding grounds and a corresponding increase in entomological inoculation rate (EIR) or infectious bites [36, 38].

Assessment of prevalence of *P. falciparum* among the enrolled children revealed that the parasites were present as asymptomatic infections in these children before the peak transmission season (Fig. 2), which corroborates a previous observation that malaria parasites are prevalent before the transmission season [5]. Harboring of parasites before the transmission season has been suggested to be protective and prevent symptomatic malaria during the peak of transmission [39] as the asymptomatic parasitaemia primes the immune system prior to any new infections. Furthermore, participants in the high transmission site had higher MOI than those in the low transmission site (Fig. 3) and this might be due to the possible increase in the frequency of infectious bites in Obom in the high transmission setting compared to Abura, the lower transmission setting [40]. Frequent infectious bites result in a greater probability of introducing new parasite clones and also higher MOI in people in high transmission settings [41]. The higher MOI observed in asymptomatic children living in Obom, the high transmission setting suggests that high transmission settings may offer increased levels of protection from symptomatic malaria due to exposure to a wide range of parasite clones during previous multi-clonal infections.

The pattern of total parasite prevalence determined by PCR in the high transmission setting, Obom was similar to that determined by microscopy. The decrease in microscopic parasite prevalence in Obom from April to May (Fig. 2a) was a result of a bednet intervention in this community [42]. Parasite prevalence observed by microscopy in children from Abura did not follow the trend of PCR detectable parasite prevalence suggesting that asymptomatic parasite carriage in this low transmission setting was mostly at submicroscopic densities. This supports a previous finding from Senegal where high proportions of asymptomatic parasite carriage in a low transmission setting during the dry season was submicroscopic [43]. A high prevalence of asymptomatic carriers may serve as a reservoir of parasites providing a continuous supply of gametocytes for transmission of the parasite [6].

Naturally acquired anti-disease immunity to malaria has been predicted to be a gradual process mediated by anti-asexual parasite immunity and more effective with increasing numbers of infecting clones [2, 3, 7]. Seroprevalence of antibodies against MSP3 in children from the high transmission setting showed monthly variations that exhibited a trend similar to that of parasite prevalence determined by both microscopy and PCR (Figs. 2b, 4), while in the low transmission settings it followed the trend of only PCR suggesting that MSP3 antibody seroprevalence in the latter settings followed that of microscopic parasite prevalence. On the other hand, seroprevalence of anti-Pfs230 antibodies followed the parasite prevalence by PCR only in Abura only (Figs. 4a, 2b). These findings support suggestions that exposure to *Plasmodium* parasites are essential in developing immunity amongst people living in endemic areas [39] and that some of the children within these communities had developed a level of immunity against symptomatic malaria [44] both before the peak malaria transmission season.

The absence of gametocytes detected after microscopic evaluation of any smear could be because most of the gametocytes in an infection are submicroscopic [20, 21]. However, the presence of antibodies to the sexual stage antigen, Pfs230, suggests a recent exposure to gametocytes [23]. Low-density infections as well as asymptomatic infections in young children and the high prevalence of asymptomatic children, which match the characteristics of children from Abura and Obom, respectively, are factors that have previously been suggested to enhance gametocyte carriage [19, 45].

Throughout the study, IgG antibody titres to Pfs230 correlated positively with those against MSP3 in both Obom and Abura (Table 3). There was very little variation in anti-MSP3 antibody responses in the children from Abura throughout the four months (Fig. 5a), suggesting the possibility of long-lived antibody responses that resulted in little or no boosting as low level infections are known to be a strategy that parasites use to evade immune activation [46].

Despite the relatively stable prevalence of children seropositive to Pfs230 during the study period in Obom, absolute antibody responses dropped from February to May (Fig. 5b) possibly due to a reduction in gametocyte densities. This can be inferred from the reduction in asexual parasite densities as antibody responses to Pfs230 have been suggested to be short-lived [23].

The absence of gametocytes in any of the samples during the microscopic examination might suggest that any gametocyte present would be at submicroscopic densities, which this study did not monitor.

## Conclusions

This study reveals that the trend of *P. falciparum* parasite prevalence variation can be predicted from antibody seroprevalence to MSP3 in asymptomatic children in the high transmission settings, while in the low transmission settings, seroprevalence to Pfs230 rather than MSP3 predicts total *P. falciparum* parasite prevalence in asymptomatic children.

## Additional files

Additional file 1: **Table S1.** *msp2* and GLURP genotyping primers. Details of the primers used for the *msp2* and GLURP genotyping reactions, including the primer name, sequence and annealing temperatures are listed in the table. (DOCX 15 kb)

Additional file 2: **Figure S1.** Representative agarose gel images of *msp*2 amplified products. Identification of the FC27 *msp2* allelic family members using the S1fw and M5 primer set (**a**) and the 3D7 *msp2* allelic family members using S1fw and N5 primer set (**b**). Lane N: no template (water) negative control; Lane C1: 3D7 positive control sample; Lanes C2: FC27 positive control sample: Lanes 1–14: samples collected in April from children living in Obom. The samples were scored as clonal or multiclonal for *msp*2 depending on the number of PCR products per sample. (DOCX 499 kb)

## Abbreviations

DBS: Filter paper blood blot
GLURP: Glutamate rich protein
MOI: Multiplicity of infection
MSP: Merozoite surface protein
OD: Optical density
PD: Parasite density
Pfs230: *Plasmodium falciparum* gametocyte surface protein P230
