# Supplementary material for: Dynamics of anti-MSP3 and Pfs230 antibody responses and multiplicity of infection in asymptomatic children from southern Ghana
Source: Parasit Vectors. 2018 Jan 5;11:13. doi: 10.1186/s13071-017-2607-5 (PMC5755320; doi:10.1186/s13071-017-2607-5)
Supplement: Supplementary file 1 — Table S1. msp2 and GLURP genotyping primers. Details of the primers used for the msp2 and GLURP genotyping reactions, including the primer name, sequence and annealing temperatures are listed in the table. (DOCX 15 kb) [file 13071_2017_2607_MOESM1_ESM.docx]

**Additional file 1: Table S1. *msp2*** and ***glurp*** genotyping primers

| **Gene/primer name** | **Primer sequence** | **Annealing temp. (^0^C)** |
| --- | --- | --- |
| ***msp2*** |  |  |
| Primary M2- OF | ATGAAGGTAATTAAAACATTGTCTATTATA | 54 |
| M2- OR | CTTTGTTACCATCGGTACATTCTT |  |
| Semi nested S1fw | GCTTATAATATGAGTATAAGGAGAA | 50 |
| 3D7 N5rev  FC27 M5rev | CTGAAGAGGTACTGGTAGA  GCATTGCCAGAACTTGAA |  |
| ***glurp*** |  |  |
| Primary G- F3 | ACATGCAAGTGTTGATCCTGAAG | 54 |
| G- F4 | TGTAGGTACCACGGGTTCTTGTGG |  |
| Nested G- NF | TGTTCACACTGAACAATTAGATTTAGATCA | 59 |
| G- F4 | TGTAGGTACCACGGGTTCTTGTGG |  |
