# Supplementary material for: Dynamics of anti-MSP3 and Pfs230 antibody responses and multiplicity of infection in asymptomatic children from southern Ghana
Source: Parasit Vectors. 2018 Jan 5;11:13. doi: 10.1186/s13071-017-2607-5 (PMC5755320; doi:10.1186/s13071-017-2607-5)
Supplement: Supplementary file 2 — Figure S1. Representative agarose gel images of msp2 amplified products. Identification of the FC27 msp2 allelic family members using the S1fw and M5 primer set (a) and the 3D7 msp2 allelic family members using S1fw and N5 primer set (b). Lane N: no template (water) negative control; Lane C1: 3D7 positive control sample; Lanes C2: FC27 positive control sample: Lanes 1–14: samples collected in April from children living in Obom. The samples were scored as clonal or multiclonal for msp2 depending on the number of PCR products per sample. (DOCX 499 kb) [file 13071_2017_2607_MOESM2_ESM.docx]

**Additional file 2: Figure S1.** Representative agarose gel images of *msp*2 amplified products. Identification of some FC27 *msp2* allelic family members using the S1fw and M5 primer set (**a**) and some 3D7 *msp2* allelic family members using S1fw and N5 primer set (**b**). Lane N: no template (water) negative control; Lane C1: 3D7 positive control sample; Lanes C2: FC27 positive control sample: Lanes 1–14: samples collected in April from children living in Obom. The samples were scored as clonal or multiclonal for *msp*2 depending on the number of PCR products produced per sample.
